# Editorial: Common Pathways Linking Neurodegenerative Diseases—The Role of Inflammation

**DOI:** 10.3389/fncel.2021.754051

**Published:** 2021-09-13

**Authors:** Małgorzata Kujawska, Andrii Domanskyi, Grzegorz Kreiner

**Affiliations:** ^1^Department of Toxicology, Poznan University of Medical Sciences, Poznań, Poland; ^2^Institute of Biotechnology, Helsinki Institute of Life Science, University of Helsinki, Helsinki, Finland; ^3^Department Brain Biochemistry, Maj Institute of Pharmacology, Polish Academy of Sciences, Kraków, Poland

**Keywords:** neurodegeneration, inflammation, oxidative stress, Parkinson's disease, Alzheimer's disease

Together with the increase in life expectancy the prevalence of neurodegenerative diseases is rapidly growing. In spite of the scale of the problem, the etiology of most neurodegenerative disorders remains unclear, and as a consequence, there are no effective treatments stopping or at least slowing down their progression. There is growing evidence that pathophysiology of different neurodegenerative diseases may overlap at multiple molecular levels and pathways ultimately leading to cell death. It is well-known, that many contributing factors i.e., inflammatory processes, oxidative damage, mitochondrial impairment, pathogenic proteins, apoptosis and autophagy dysfunction—all well-established features of neurodegeneration—are not exclusively related to any particular neurodegenerative disorder and identified in both sporadic and familial forms (Ramanan and Saykin, 2013; Ruffini et al., 2020). Oxidative stress and inflammation, which are tightly linked and interdependent, are regarded as playing a key role in neurodegeneration pathogenesis. This is why maintaining the redox balance, based on the generation and elimination of reactive oxygen species by endogenous and exogenous sources, is crucial for neuroprotection. Similarly, exposure to various environmental factors, such as pesticides, are known contributors to the increased risk of age-related neurodegeneration (Cannon and Greenamyre, 2011; Baltazar et al., 2014). As pointed out in review by Kamal et al., the most prevalent neurodegenerative diseases like Alzheimer's and Parkinson's disease can be also associated with alcohol dependence as prolonged alcohol intake contributes to production of reactive oxygen species triggering neuroimmune response and neural cell death. While it is generally possible—and, in view of current scientific evidence, also highly advisable—to avoid prolonged alcohol intake and thus prevent neurodegeneration (and multiple other diseases), it is much more difficult to avoid the exposure to polluted air. In their article, Jankowska-Kiełtyka et al. review published evidence demonstrating the role of air pollution as a contributing factor to onset and progression of neurodegeneration, highlighting increased oxidative stress and inflammation as possible molecular mechanisms linking exposure to particular matter with neuronal death. With more research done in recent years, it becomes increasingly evident that neurodegenerative diseases arise as a result of complex interplay between aging, genetic, lifestyle and environmental factors, affecting multiple cell types, with inflammation playing a crucial role in the process. For example, spreading inflammation has been observed in Alzheimer's disease. Since evidence supports the inflammatory crosstalk between the periphery and the central nervous system *via* the blood-brain barrier (BBB), Ni and Wu show that the “dampening inflammation,” particularly through the inhibition of cathepsin, appears to be a novel therapeutic approach delaying the onset of and enacting early intervention for Alzheimer's disease.

Understanding the neural networks and their involvement in neurodegenerative conditions could provide better insights into the principles of neurodegeneration. As summarized by Afridi et al. age-related chronic inflammatory activation of microglia and their dysfunction can be considered as one of pivotal insults as the housekeeping and defensive functions of microglia are of particular importance for brain homeostasis. Furthermore, the authors conclude that dysfunctional microglia may directly contribute to phagocytosis, increased pro-inflammatory cytokine secretion and reactive oxygen species release. It is also important to bear in mind the complexity and heterogeneity of microglia, reflected by their morphology and expression of cellular markers reflecting functional differences between different microglial phenotypes. As reviewed by Jurga et al., during progression of neurodegeneration, different types of microglia can have either pro-inflammatory or anti-inflammatory functions and it is critical to understand the contribution of these microglia phenotypes to disease progression in order to develop new therapeutic strategies targeting microglia. While microglial dysfunction may arise in part as a result of lipid dyshomeostasis, phosphoinositides (PiPs) appear to be key players in regulating microglial-mediated neuroinflammation. PIPs, as reviewed by Phillips and Maguire, regulate the activities of proteins and enzymes required for endocytosis, toll-like receptor signaling, purinergic signaling, chemotaxis, and migration, all of which are affected in a variety of neurodegenerative conditions. Given the importance of microglia and PIPs in dementia development, possible future therapeutic approaches would benefit from their targeted co-manipulation.

Inflammatory response can be activated by stimulation of toll-like receptors (TLRs), resulting in changes in morphology and production and release of cytokines which was the topic of interest for Gilchrist et al. In this paper the authors examined the roles of the related TAM receptors, Mer and Axl, and of their ligand, Gas6, in the regulation of microglial induced pro-inflammatory response, providing evidence that Gas6 may negatively regulate the detrimental response to lipopolysaccharide (LPS) as well as *via* stimulation of other TLRs. Therefore, as concluded by authors of this work, anti-inflammatory role for the TAM ligand, Gas6, could be of particular interest due to therapeutic potential.

The inflammatory response in animal models is often modeled by administration of LPS, which was also implemented in *in-vitro* study on PC12 cells presented by Sangaran et al. In this paper, the authors proposed neuroprotective effect *via* pre-activation of toll-like receptor-4 signaling pathway leading to the inhibition of Caspase-3/nuclear factor-kappaB (NF-κB) pathway. The role of NF-κB, a transcription factor involved in the activation of the inflammasome was reviewed in context of cerebral ischemia by Jover-Mengual et al. Growing evidence indicates a dual role of inflammation in cerebral ischemia. While the acute inflammatory response aggravates an ischemic injury, the recovery and tissue repair depend on later inflammatory processes. Therefore, according to the authors, effective therapy should selectively inhibit the deleterious constituents of the inflammatory process during cerebral ischemia and simultaneously enhance the beneficial aspects of the inflammatory response. What is of particular importance, the authors fairly stated all pros and cons regarding the role of NF-κB in neurodegeneration which is controversial whether its activation promotes neuroprotective response or—as recently more supported—rather contributes to inflammation and neural cell death. It is worth to mention that NF-κB has been implicated in the pathogenesis of a number of autoimmune diseases as well. One of the most popular example is multiple sclerosis (MS) where NF-κB was confirmed to be activated in micro-/astroglia and neurons. MS is mostly studied in experimental autoimmune encephalomyelitis (EAE) model which was the topic pursued by Nam et al. who showed that administration of glucocorticoids and mesencephalic astrocyte-derived neurotrophic factor (MANF) in EAE mice improved in similar way the phenotype in the early stage of disease. Certain aspects of MS can also be modeled in mouse hepatitis virus (MHV)-induced chronic neuroinflammatory demyelination. Using this model, Sarkar et al. have demonstrated that *Azadirachta indica* (Neem) bark extract suppresses MHV-induced neuroinflammation and neuropathogenesis by inhibiting cell-to-cell fusion and viral replication. Moreover, the authors suggested antiviral activity of Neem extract against other Spike expressing human coronaviruses like SARS-CoV-2.

MANF and closely related cerebral dopamine neurotrophic factor (CDNF), as well as glial cell line-derived neurotrophic factor (GDNF), neurturin (NRTN), and PDGF-BB, supporting the development, maturation, and survival of neurons demonstrate potential to rescue and regenerate neuronal populations lost in neurodegenerative diseases. Indeed, as reviewed by Kotliarova and Sidorova, available data strongly support the ability of GDNF family ligands (GFLs) to influence both neuronal and glial cells, which increase GFLs attractiveness of being a target for developing novel treatments against neurodegeneration. However, neurotrophic factors do not pass through the BBB and are delivered directly into patients' brains using costly and risky intracranial surgery, which is also restricting their application to patients at the late stage of neurodegeneration, essentially limiting the effectiveness of such therapy. As reviewed by Bondarenko and Saarma, nanoparticles (NPs) with the size dimensions of 1–100 nm can be used to facilitate their transport through the BBB. As NPs themselves can prevent the aggregation of proteins, reduce inflammation, and alleviate stress, the dual strategy combining drug carrier and therapeutic function of NPs may be of significance, especially for treating protein-aggregation-related neurodegenerative disorders.

The role of oxidative stress, inflammation and mitochondrial dysfunction is by no means limited only to “classical” neurodegenerative diseases. Age-related macular degeneration (AMD), which most commonly originates from choroidal neovascularization (CNV), is the leading cause of blindness in the elderly. The newly released study by Kim et al. demonstrates that early microglial matrix metalloproteinase-9 (MMP-9) activation contributes to CNV. Thus, the authors suggest modulation of microglial MMP expression as a novel putative therapeutic target for CNV. The results of Zuo et al. show that treatment with elamipretide, a mitochondrial-targeted peptide, seems to be a promising strategy for treating perioperative neurocognitive disorders associated with mitochondrial dysfunction and pyroptosis.

Our understanding of molecular mechanisms driving neurodegeneration heavily relies on the use of animal models. Despite significant progress in studies using rodents, development of new models better recapitulating the onset and progression of human diseases, as well as tools to characterize these models is desperately needed. An interesting study by Spellicy et al. using a semi-auto high content imaging (HCI) and CellProfiler analysis approach to analyze histopathological features following ischemic stroke. The authors have revealed regional and cell-specific morphological signatures of immune and neural cells after stroke in a highly translational porcine model, showing remarkable similarity to the human stroke condition. This unbiased and sensitive tool to quantify different cells' morphological changes and interaction between cells in the stroke environment offers a uniquely therapeutic and biomarker discovery opportunity. Another rapidly emerging alternative model organism used in neuroscience is zebrafish (*Danio rerio*). Utilizing cutting-edge CRISPR/Cas9 technology, Korzeniowska et al. successfully edited zebrafish genome introducing mutation in NPC2 gene, causing Niemann-Pick type C (NPC) disease in humans. NPC is a rear lipid storage disorder characterized by abnormal accumulation of lipids in multiple organs, including the brain. As stated on Orphanet (a unique resource, gathering and improving knowledge on rare diseases) website rare diseases are rare but rare diseases patients are numerous. The authors demonstrate that their model can faithfully reproduce neurological and inflammatory symptoms of NPC disease and therefore may be used for testing novel therapeutic strategies in preclinical studies.

Overall, in this Research Topic, we have gathered 17 articles summarizing various aspects of mechanisms behind neurodegeneration studied in cellular and animal models. These were not particularly focusing on specific disease and the neural populations directly responsible for symptomatology, but rather more general, linking i.e., the immune system and oxidative stress in brain aging and neurodegeneration, glial responses and neuroinflammation in neurodegenerative diseases, innate immune system and neurodegeneration.

Altogether, articles in this Research Topic clearly demonstrate the importance of oxidative stress and inflammation in neurodegenerative diseases. It is becoming increasingly appreciated that progression of neurodegeneration is also affected by complex interactions between neural cells and microglia, and the cellular pathways modulated by such interactions are being deciphered. Identifying particular proteins and pathways as convergence points in developing neurodegenerative diseases alongside unbiased and sensitive assessment methods is fundamental for understanding their causes and mechanisms, offering novel therapeutic and biomarker discovery opportunities.

## Author Contributions

All authors listed have made a substantial, direct and intellectual contribution to the work, and approved it for publication.

## Funding

This research was funded by the Academy of Finland (#293392, #319195), Päivikki and Sakari Sohlberg Foundation and NCN grant no 2017/25/B/NZ7/02406 Opus13.

## Conflict of Interest

The authors declare that the research was conducted in the absence of any commercial or financial relationships that could be construed as a potential conflict of interest.

## Publisher's Note

All claims expressed in this article are solely those of the authors and do not necessarily represent those of their affiliated organizations, or those of the publisher, the editors and the reviewers. Any product that may be evaluated in this article, or claim that may be made by its manufacturer, is not guaranteed or endorsed by the publisher.
